# Development and validation of a nomogram for predicting atrial fibrillation in patients with acute heart failure admitted to the ICU: a retrospective cohort study

**DOI:** 10.1186/s12872-022-02973-3

**Published:** 2022-12-06

**Authors:** Yide Li, Zhixiong Cai, Yingfang She, Wenjuan Shen, Tinghuai Wang, Liang Luo

**Affiliations:** 1 Department of Critical Care Medicine, The Seventh Affiliated Hospital, Sun Yat-Sen University, Shenzhen, China; 2 Department of Cardiology, Shantou Central Hospital, Shantou, China; 3 Neurology Medicine Center, The Seventh Affiliated Hospital, Sun Yat-Sen University, Shenzhen, China; 4 Department of Physiology, Zhong Shan School of Medicine, Sun Yat-Sen University, Guangzhou, China

**Keywords:** Nomogram, Acute heart failure, Atrial fibrillation, Prediction model, Disease severity

## Abstract

**Introduction:**

Acute heart failure is a serious condition. Atrial fibrillation is the most frequent arrhythmia in patients with acute heart failure. The occurrence of atrial fibrillation in heart failure patients worsens their prognosis and leads to a substantial increase in treatment costs. There is no tool that can effectively predict the onset of atrial fibrillation in patients with acute heart failure in the ICU currently.

**Materials and methods:**

We retrospectively analyzed the MIMIC-IV database of patients admitted to the intensive care unit (ICU) for acute heart failure and who were initially sinus rhythm. Data on demographics, comorbidities, laboratory findings, vital signs, and treatment were extracted. The cohort was divided into a training set and a validation set. Variables selected by LASSO regression and multivariate logistic regression in the training set were used to develop a model for predicting the occurrence of atrial fibrillation in acute heart failure in the ICU. A nomogram was drawn and an online calculator was developed. The discrimination and calibration of the model was evaluated. The performance of the model was tested using the validation set.

**Results:**

This study included 2342 patients with acute heart failure, 646 of whom developed atrial fibrillation during their ICU stay. Using LASSO and multiple logistic regression, we selected six significant variables: age, prothrombin time, heart rate, use of vasoactive drugs within 24 h, Sequential Organ Failure Assessment (SOFA) score, and Acute Physiology Score (APS) III. The C-index of the model was 0.700 (95% CI 0.672–0.727) and 0.682 (95% CI 0.639–0.725) in the training and validation sets, respectively. The calibration curves also performed well in both sets.

**Conclusion:**

We developed a simple and effective model for predicting atrial fibrillation in patients with acute heart failure in the ICU.

**Supplementary Information:**

The online version contains supplementary material available at 10.1186/s12872-022-02973-3.

## Introduction

The global burden of disease-related heart failure is increasing since it is a condition that generally cannot be cured. In 2015, there were an estimated 40 million heart failure patients worldwide [1]. In the population over 70 years of age, the prevalence may be greater than 10% [2]. In the United States alone, heart failure accounts for $39 billion of annual health care costs [3]. It is the leading cause of hospitalization among older adults [4]. The majority of patients are admitted with acute heart failure when the signs and symptoms change rapidly enough to require the acquisition of medical care. Of these patients, more than 10% need to be admitted to the intensive care unit (ICU) [5]. Additionally, the prognosis in these patients is dismal, with in-hospital mortality ranging from 4 to 10% [6–8], and one-year mortality ranging from 10 to 36% [9–12].

Atrial fibrillation is the most common arrhythmia in patients with acute heart failure, with a prevalence between 30 and 45% [12–14]. It may be associated with the same etiology that caused acute heart failure, or it may be prompted by the development of pathophysiological changes occurring during acute heart failure [15]. Atrial fibrillation is one of the risk factors for increased all-cause mortality in patients with acute heart failure [16, 17], and contributes to a significantly increased risk of thrombotic events [18, 19]. The risk factors for atrial fibrillation have been adequately studied for decades [20, 21], and a few predictive models have also been developed [22, 23]. Nevertheless, the performance of the model is plain [24, 25]. There is currently no predictive model for atrial fibrillation onset in heart failure patients that is applicable to the ICU setting. The objective of this study was to develop and validate a tool that could effectively predict the probability of atrial fibrillation in patients admitted to the ICU for acute heart failure.

## Materials and methods

### Database

MIMIC-IV is a relational database containing real hospitalization data of patients admitted to a tertiary medical center in Boston, MA, USA [26, 27]. It contains data of 76,540 ICU stays from 2008 to 2019. The database is de-identified, and patient identifications were removed according to the Health Insurance Portability and Accountability Act Safe Harbor provision. The database contains complete information regarding the hospital stay of each patient and was designed to support various researches in the medical field. The latest version is v1.0 (March 16th, 2021). In this study, we used the current version of the database, and PostgreSQL v11.1 was used to search and extract the data (http://www.postgresql.org/).

Because this study was a retrospective analysis of data stored in a publicly available database, the institutional review board approval was exempted and patient consent was not required.

### Study populations

This is a retrospective analysis of the data of adult patients with acute heart failure admitted to the ICU, which are stored in the MIMIC-IV database. The inclusion criteria were as follows:

1. Patients with a diagnosis of ‘acute heart failure’ (Specific ICD codes are available in the Additional file 1: Table S1).
2. Patients who were admitted to the ICU for the first time.
3. Patients aged older than 18 years.
4. Patients whose length of ICU stay was more than 48 h

### Data collection

The characteristics extracted for our study included demographic data (e.g., age and gender), Charlson comorbidity index, disease severity scores (SOFA and APSIII), vital signs (e.g., heart rate and blood pressure), cardiac rhythm records, urine output, dialysis activated, mechanical ventilation, laboratory results (e.g., blood routine test, chemistry, coagulation, cardiac marker) and vasoactive agents (dopamine, epinephrine, norepinephrine, phenylephrine, vasopressin, dobutamine, and milrinone) used. Data on all the characteristics were collected between 6 h before and 24 h after ICU admission. The endpoint event was the onset of atrial fibrillation during the ICU stay.

### Statistical analysis

Statistical analysis was performed using the R software (Version 4.1.3; R Foundation for Statistical Computing, Vienna, Austria; https://www.r-project.org). The distribution of continuous variables was assessed using the Shapiro–Wilk test. Mean and standard deviation (SD) are reported for continuous variables having a normal distribution; median and interquartile range (IQR), for continuous variables having a skewed distribution; and numbers and percentages, for categorical variables. The Kruskal–Wallis H test was used to compare continuous data. The Fisher’s exact test was used to compare categorical variables. Seventy percent of the data was allocated to the training set for developing the model, and the remaining 30% was allocated for validation. Multiple imputations using chained equations were adopted to replace the missing values. The least absolute shrinkage and selection operator (LASSO) method, which accounts for multicollinearity and avoids model overfit, was used to select the optimal candidate variable for constructing the nomogram [28]. To avoid making the clinical prediction model too complex, the lambda value was set to ‘1se’ so that the error was within one standard error of the minimum. Sensitivity and specificity were used to evaluate the model’s performance. The calibration C index (bootstrap resampling performed 1000 times), the calibration curve, Hosmer–Lemeshow goodness-of-fit test, and receiver operating characteristic (ROC) curves were used to evaluate the discrimination and calibration of the model. Decision curve analysis (DCA) was used to evaluate the clinical usefulness of the model. All significance tests were two-sided, and a *P* value < 0.05 was considered statistically significant.

## Results

### Study population

**Fig. 1:**
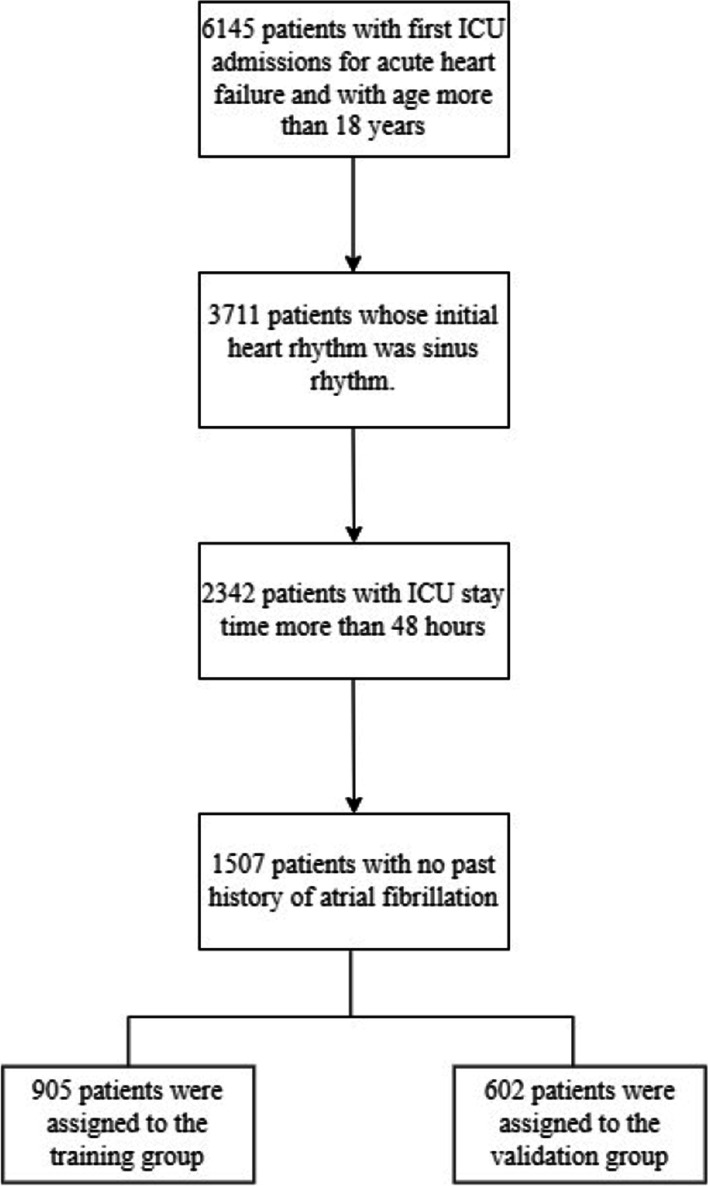
Flowchart showing the process of cohort selection

Figure 1 illustrates the cohort selection process. In total, 2342 patients were eligible for the study. Among them, 70% (1641 patients), were assigned to the training set and the rest were assigned to the validation set (demographics for the two sets are shown in the Additional file 1: Table S2).

Table 1 demonstrates the patients’ demographic baseline characteristics. Of the 2342 patients who initially had sinus rhythm, 646 (27.58%) developed atrial fibrillations during their ICU stay. Patients in the atrial fibrillation group were older and had higher Charlson comorbidity scores, along with higher in-hospital mortality and longer ICU stays.

**Table 1:** The demographics of the cohort

	Overall	No-AF group	AF group	*P*
Numbers	2342	1696	646	
Male (%)	1216 (51.9)	851 (50.2)	365 (56.5)	0.007
Age (year), median [IQR]	71.65 [61.24, 81.50]	69.72 [59.01, 80.36]	75.50 [67.11, 83.40]	< 0.001
Ethnicity (%)				0.002
American Indian/Alaska native	7 (0.3)	6 (0.4)	1 (0.2)	
Asian	55 (2.3)	38 (2.2)	17 (2.6)	
Black/African American	245 (10.5)	202 (11.9)	43 (6.7)	
Hispanic/Latino	73 (3.1)	57 (3.4)	16 (2.5)	
Other	96 (4.1)	79 (4.7)	17 (2.6)	
Unable to obtain	33 (1.4)	24 (1.4)	9 (1.4)	
Unknown	310 (13.2)	221 (13.0)	89 (13.8)	
White	1523 (65.0)	1069 (63.0)	454 (70.3)	
Length of hospital stay (day), median [IQR]	11.07 [7.21, 17.04]	10.20 [6.84, 16.43]	12.96 [8.73, 19.06]	< 0.001
Hospital mortality (%)	292 (12.5)	181 (10.7)	111 (17.2)	< 0.001
Length of ICU stay(day), median [IQR]	4.15 [2.90, 6.92]	3.82 [2.71, 5.85]	5.81 [3.67, 9.91]	< 0.001
Charlson comorbidity index, median [IQR]	7.00 [6.00, 9.00]	7.00 [5.00, 9.00]	8.00 [6.00, 9.00]	< 0.001
SOFA score, median [IQR]	6.00 [4.00, 9.00]	6.00 [3.00, 8.00]	7.00 [5.00, 10.00]	< 0.001
APS III, mean (SD)	53.25 (23.99)	50.78 (22.92)	59.73 (25.52)	< 0.001
Mean blood pressure(mmHg), median [IQR]	75.07 [69.21, 82.13]	75.56 [69.48, 82.92]	73.76 [68.82, 80.10]	< 0.001
Heart rate, median [IQR]	84.51 [74.10, 95.20]	83.86 [73.72, 94.61]	85.92 [75.79, 96.92]	0.009
Temperature (℃), median [IQR]	36.80 [36.57, 37.11]	36.82 [36.58, 37.13]	36.76 [36.53, 37.05]	0.001
SpO2(%), median [IQR]	96.64 [95.08, 98.12]	96.57 [95.09, 98.03]	96.81 [95.03, 98.32]	0.184
Respiratory rate, median [IQR]	13.00 [11.00, 16.00]	13.00 [11.00, 16.00]	13.00 [10.00, 15.00]	0.065
White blood cell (*109/L), (median [IQR])	11.80 [8.65, 15.60]	11.45 [8.45, 15.30]	12.47 [9.26, 16.75]	< 0.001
Hemoglobin (g/dl), median [IQR]	10.55 [9.10, 12.20]	10.55 [9.10, 12.20]	10.55 [9.10, 12.09]	0.65
Hematocrit (%), mean (SD)	32.30 [28.00, 37.30]	32.30 [28.00, 37.45]	32.15 [28.01, 37.12]	0.442
Albumin (g/dl), median [IQR]	3.30 [2.90, 3.70]	3.30 [2.90, 3.70]	3.30 [2.90, 3.69]	0.608
Anion gap (mmol/L), median [IQR]	15.50 [13.00, 17.50]	15.00 [13.00, 17.50]	15.50 [13.50, 18.00]	0.079
HCO3-(mmol/L), median [IQR]	23.00 [20.38, 26.00]	23.50 [20.50, 26.50]	22.50 [20.00, 25.50]	< 0.001
BUN (mg/dl), median [IQR]	27.50 [18.50, 44.00]	26.50 [18.00, 42.25]	30.00 [19.50, 49.38]	< 0.001
Creatine (mg/dl), median [IQR]	1.25 [0.90, 2.00]	1.25 [0.90, 1.95]	1.35 [0.95, 2.15]	0.011
Calcium(mmol/L), median [IQR]	8.45 [8.00, 8.85]	8.45 [8.00, 8.90]	8.35 [7.95, 8.75]	< 0.001
Blood glucose (mg/dl), median [IQR]	142.50 [115.50, 183.50]	141.00 [115.00, 182.50]	147.25 [117.00, 189.50]	0.059
Sodium (mmol/L), median [IQR]	138.00 [135.50, 140.50]	138.00 [135.50, 140.50]	138.00 [135.12, 140.50]	0.715
Potassium (mmol/L), median [IQR]	4.25 [3.90, 4.70]	4.20 [3.90, 4.65]	4.30 [4.00, 4.80]	< 0.001
PT(s), median [IQR]	14.00 [12.50, 16.40]	13.70 [12.40, 15.85]	14.75 [12.90, 17.60]	< 0.001
PTT(s), mean (SD)	45.02 (24.22)	44.54 (24.45)	46.23 (23.63)	0.14
Alanine aminotransferase(U/L), median [IQR]	32.00 [18.00, 75.50]	32.00 [18.00, 68.00]	33.00 [17.38, 94.25]	0.425
Aspartate aminotransferase(U/L), median [IQR]	45.50 [27.50, 122.50]	45.00 [27.00, 109.00]	49.25 [28.00, 147.00]	0.076
Total bilirubin(mg/dL), median [IQR]	0.65 [0.40, 1.10]	0.60 [0.40, 1.10]	0.65 [0.40, 1.10]	0.323
Troponin-T (ng/ml), median [IQR]	0.28 [0.07, 1.24]	0.29 [0.07, 1.27]	0.26 [0.07, 1.18]	0.559
NT-proBNP (pg/ml), median [IQR]	5168.00 [2058.50, 13,115.75]	4838.50 [1986.50, 12,914.50]	6080.00 [2449.75, 13,581.50]	0.132
Dialysis activated within 24 h in ICU (%)	69 (2.9)	49 (2.9)	20 (3.1)	0.898
Urine output(ml/kg) (median [IQR])	0.84 [0.49, 1.38]	0.89 [0.52, 1.43]	0.72 [0.41, 1.22]	< 0.001
The usage of the vasoactive agent in the first 24 h, (%)	1030 (44.0)	669 (39.4)	361 (55.9)	< 0.001
Mechanical ventilation within the first 24 h in ICU (%)	1042 (44.5)	706 (41.6)	336 (52.0)	< 0.001

*APS III* Acute Physiology Score III; *BPM* beats per minute; *BUN* Blood urea nitrogen; *ICU* intensive care unit; *IQR* interquartile range; *NT-proBNP* N-terminal pro-B-type natriuretic peptide; *PT* Prothrombin time; *PTT* Partial thromboplastin time; *SD*: standard deviation; *SOFA* score: Sequential Organ Failure Assessment

### Variable selection

**Fig. 2:**
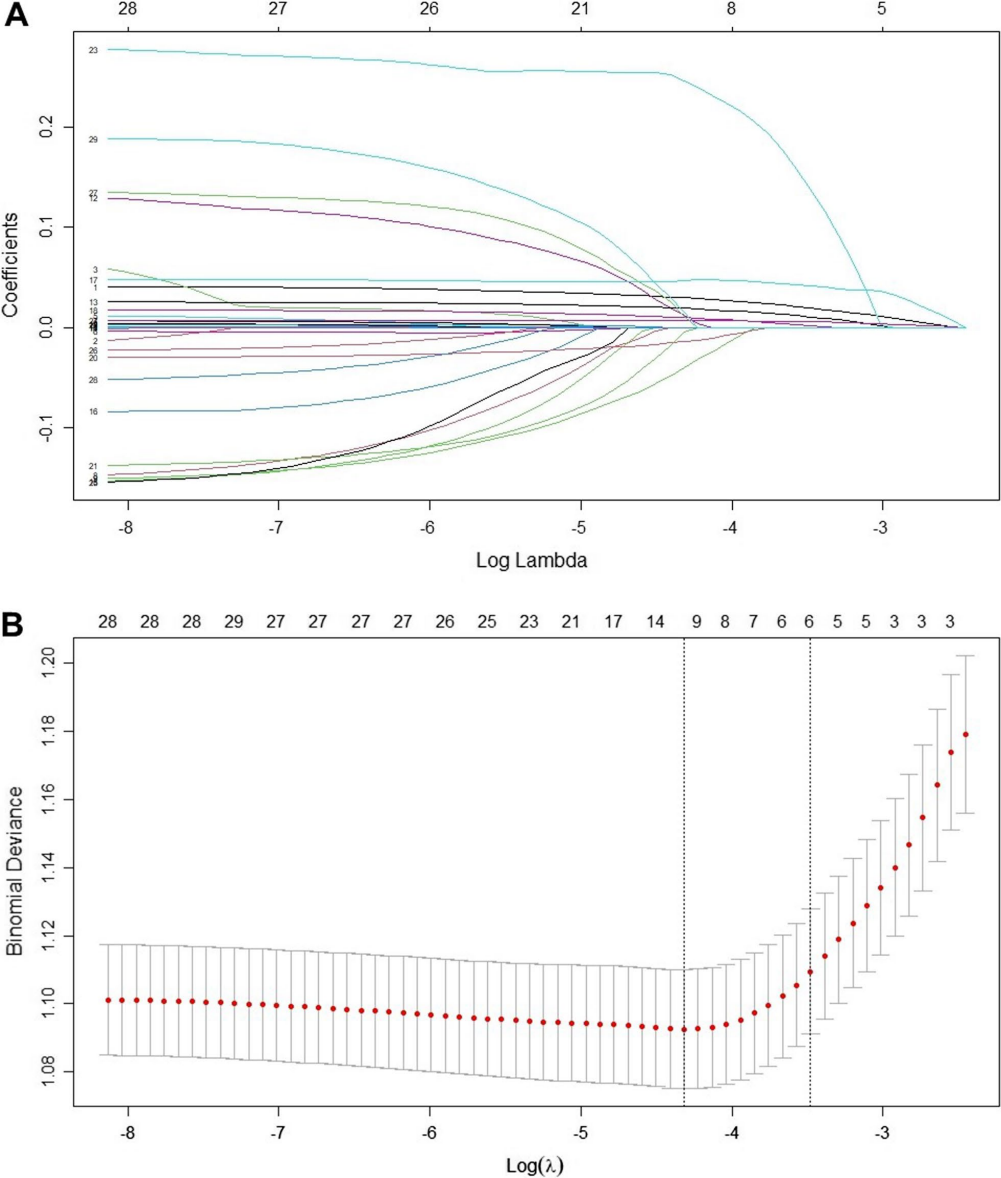
Variable selection using LASSO regression. **A** LASSO coefficient profiles of the 29 candidate variables. **B** Turing parameter (λ) selection in the LASSO model using tenfold cross-validation. LASSO: least absolute shrinkage and selection operator

**Fig. 3:**
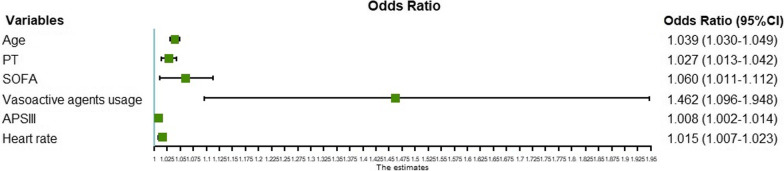
Multivariate analysis of atrial fibrillation. *APS* acute physiology score; *CI* confidence interval; *PT* prothrombin time; *SOFA* sequential organ failure assessment

Among the 33 candidate variables, the values of liver function tests (alanine aminotransferase, aspartate aminotransferase, total bilirubin, albumin) were missing for 20 to 40% of the patients. These variables were not included in the regression analysis because of uncertainty in imputation due to the high proportion of missing values and the scarcity of literature showing their correlation with arrhythmias. The values of troponin-T and N-terminal pro-b-type natriuretic peptide were missing in more than 20% and 40% of patients, respectively, but as they are important parameters for assessing the cardiac function, flags indicating whether these tests were obtained were used as a variable. The missing values of all other variables were less than 5% (detailed information can be found in the Additional file 1: Table S3). Six variables were finally screened using tenfold cross-validated LASSO regression: age, prothrombin time (PT), Sequential Organ Failure Assessment (SOFA) score, heart rate, usage of vasoactive drugs, and Acute Physiology Score (APS) III. Figure 2 illustrates the variable selection process. These six variables were included in the multivariate logistic regression model for predicting the occurrence of atrial fibrillation, and their odd ratios and 95% confidence intervals (CIs) are shown in Fig. 3.

### Construction of a predicting nomogram

**Fig. 4:**
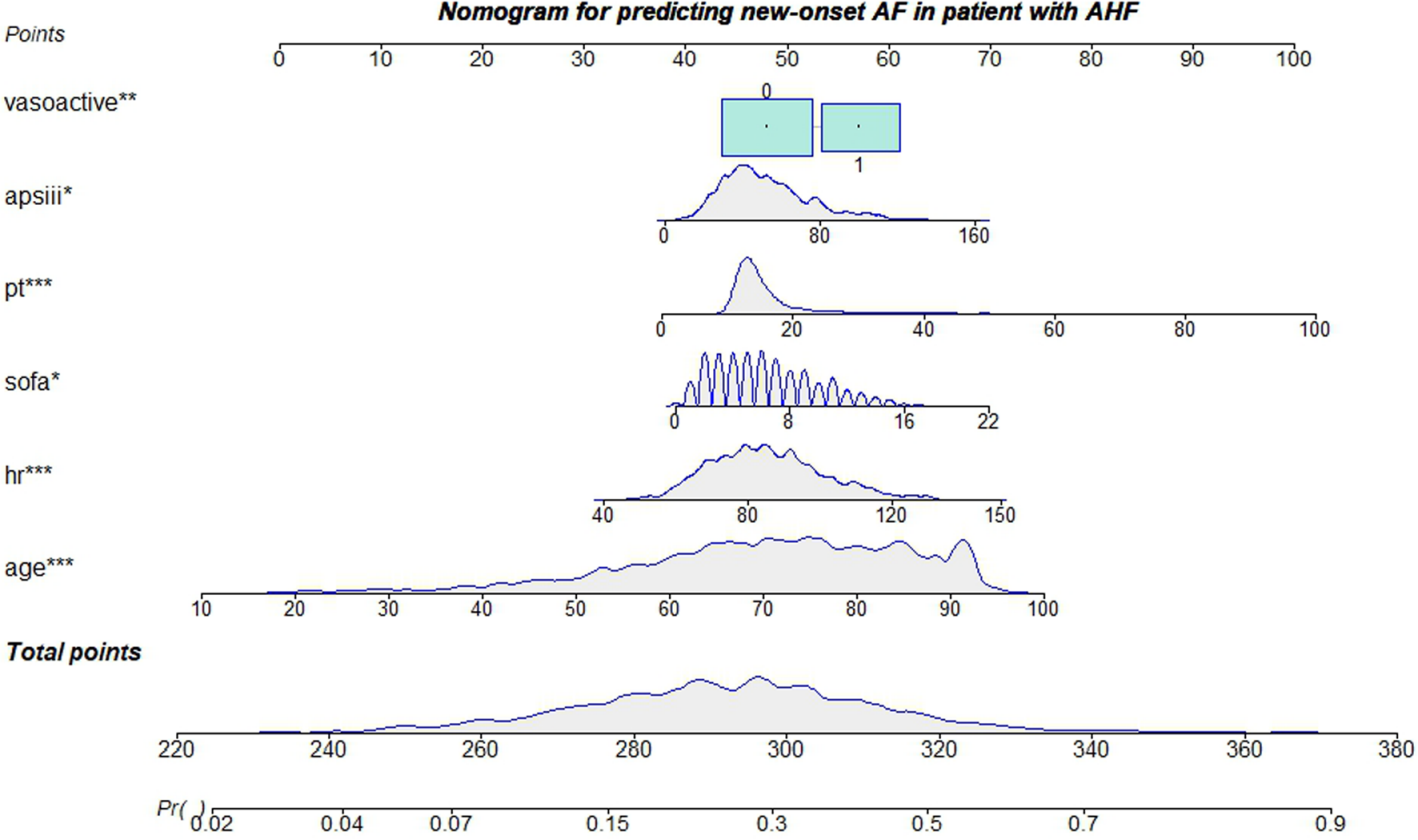
Nomogram for predicting atrial fibrillation in patients with acute heart failure. *: *P* < 0.05; **: *P* < 0.01; ***: *P* < 0.001, AF: atrial fibrillation; *AHF* acute heart failure; apsiii: Acute Physiology Score III; *hr* heart rate; *pt* prothrombin time; *sofa* Sequential Organ Failure Assessment

A nomogram was developed for predicting the occurrence of atrial fibrillation among patients in the ICU (Fig. 4). The six variables mentioned above were assigned different initial scores and a total score was calculated. The higher the total score, the higher the probability of atrial fibrillation.

### Assessment and validation of the nomogram

**Fig. 5:**
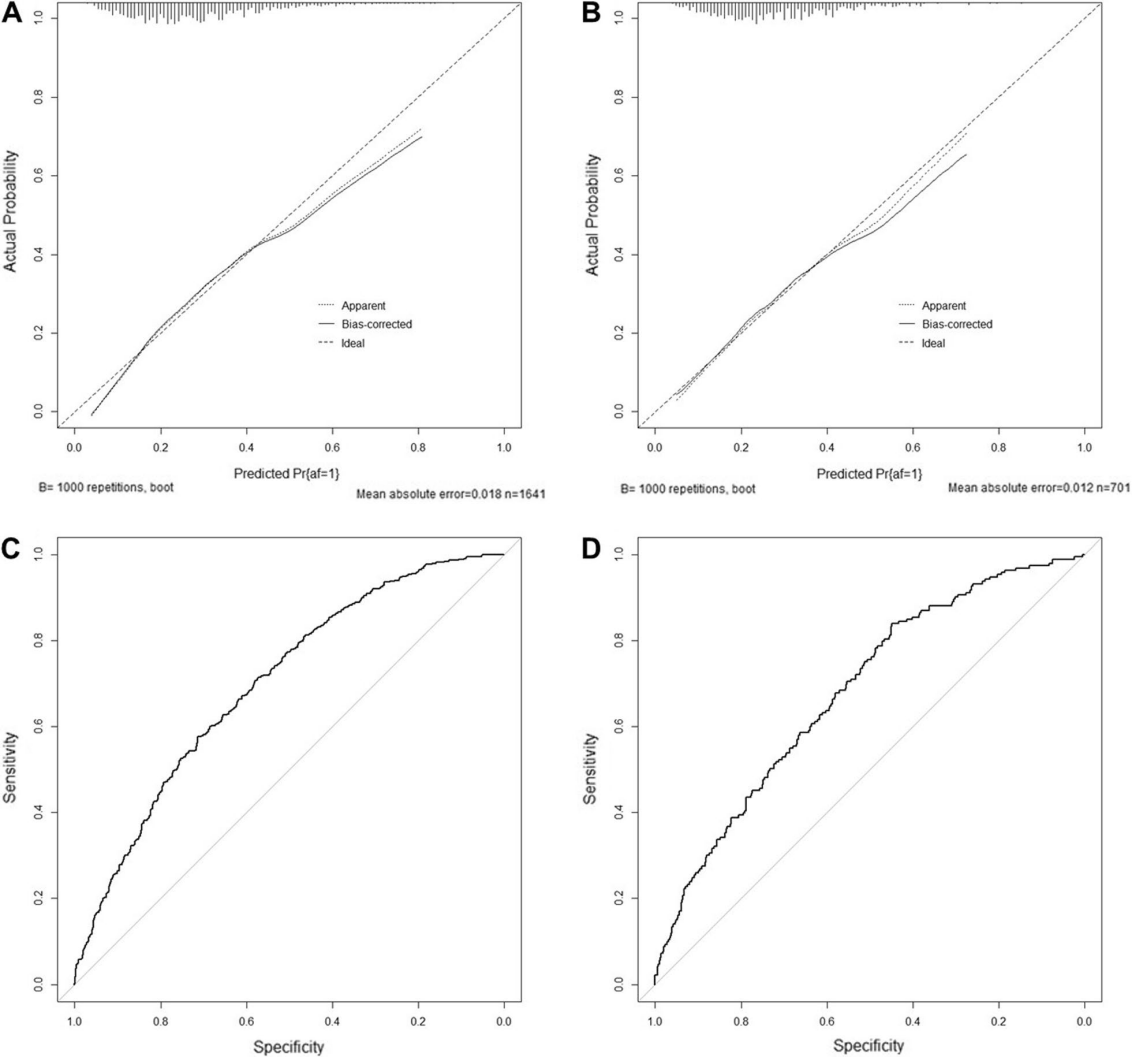
Assessment and Validation of the Nomogram. (**A**) Calibration plot for the training set and (**B**) and validation set. (**C**) Receiver operating characteristic curve of the training set and (**D**) and validation set

The Hosmer–Lemeshow test showed high conformity between the predicted and observed probabilities for both the training set (*P* = 0.441) and the validation set (*P* = 0.762). The two calibration curves demonstrate the same conclusion (Fig. 5A and B). The area under the ROC curve was 0.700 (95% CI 0.672–0.727) in the training set and 0.682 (95% CI 0.639–0.725) in the validation set (Fig. 5C and D).

**Fig. 6:**
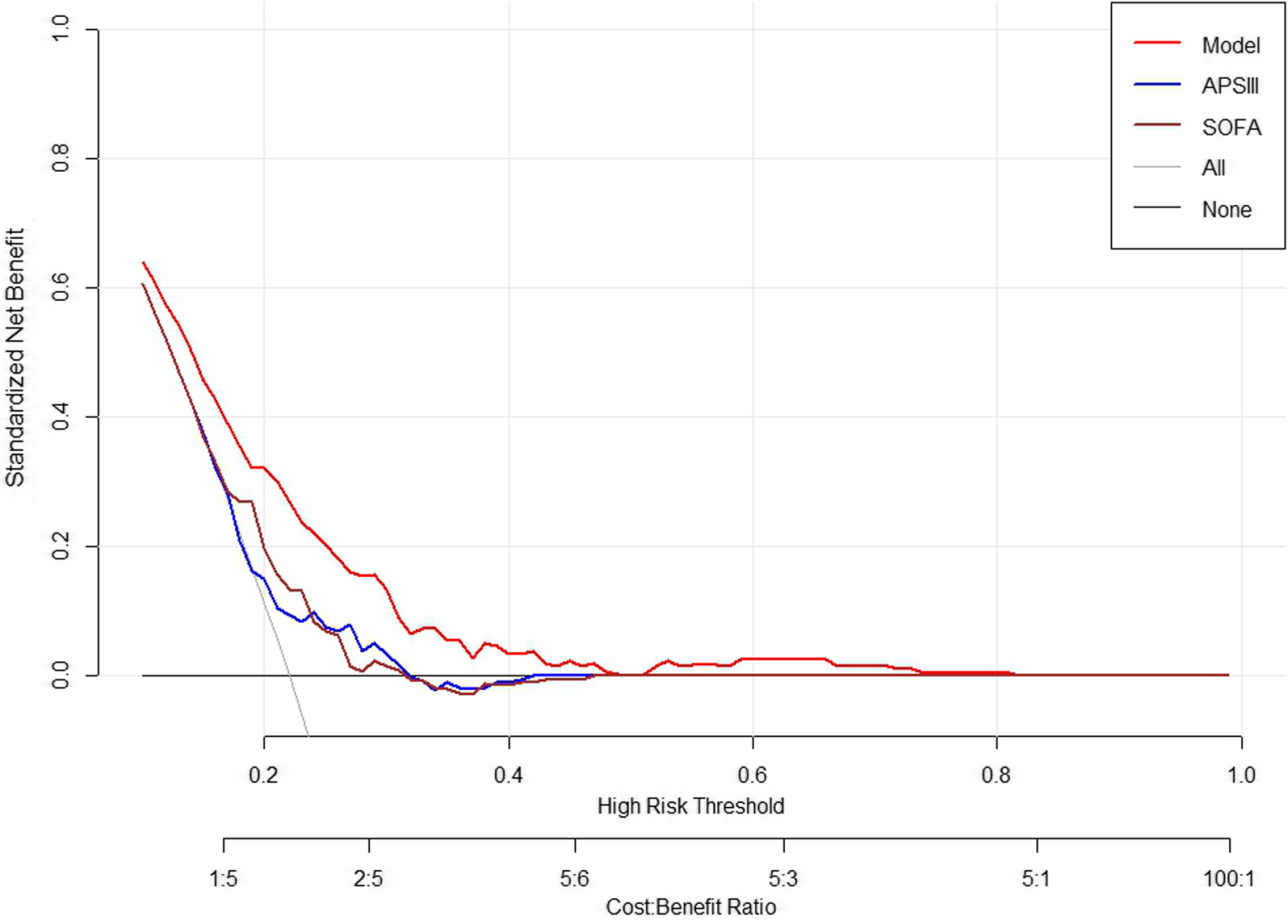
Decision curve analysis for predicting atrial fibrillation. Our model outperformed the other two scores. *APS III* Acute Physiology Score III; *SOFA* Sequential Organ Failure Assessment

DCA showed that the clinical benefit of our model was more pronounced when compared with that of the other two disease severity scores (SOFA and APS III) separately (Fig. 6).

To facilitate the application of our model in clinical practice, we developed an online web calculator to calculate the risk of atrial fibrillation for patients with acute heart failure in the ICU (https://fragilelife.shinyapps.io/AFinAHF/).

## Discussion

In this study, we retrospectively analyzed the data of 2342 patients admitted to the ICU with acute heart failure. The incidence of atrial fibrillation in the ICU was 27.58%. A model to predict atrial fibrillation was developed and validated. The nomogram contained six variables and displayed good discrimination and calibration in both the training and validation sets.

Acute heart failure and atrial fibrillation often coexist and are mutually dependent. Atrial fibrillation is the most frequent arrhythmia occurring in patients with heart failure and may have several causes. Heart failure and atrial fibrillation share many common risk factors: such as hypertension, smoking, obesity, sleep apnea syndrome, coronary artery disease, and diabetes mellitus [29]. In heart failure, the increased intra-atrial pressure and dilated atria both lead to aberrant electrical activity and predispose the patient to atrial fibrillation [30]. Neurohormonal changes in heart failure enable the development and maintenance of atrial fibrillation. Previous studies have confirmed that the activation of mitogen-activated protein kinase and increase in angiotensin II levels in heart failure contribute to the activation of arrhythmogenic atrial structural remodelling [31]. After the onset of atrial fibrillation, up to 25% of cardiac output can be lost owing to the loss of atrial diastolic function, and the rapid ventricular rate can also cause impaired left heart function [32]. Therefore, management of heart failure becomes even more difficult. This study identified six variables that predicted atrial fibrillation in acute heart failure, all of which are readily available in regular clinical practice and can be assessed easily.

The use of vasoactive drugs depends on the physician's assessment of the patient’s hemodynamic status to determine the presence of shock rather than a single measurement of low blood pressure. The causes of atrial fibrillation differ in different types of shock. In septic shock, atrial fibrillation may occur due to a systemic inflammatory response [33, 34]. In cardiogenic shock, the rapid dilatation of the atria and the increase in atrial mechanical pressures induce electrophysiological disturbances that lead to atrial fibrillation [35]. Hypovolemia can directly interfere with normal electrical conduction, which can cause atrial fibrillation [17]. In obstructive shock typified by pulmonary embolism, a dramatic increase in pulmonary artery pressure, stretch injury to right atrial tissue, right ventricular dilatation, and volume overload can all contribute to the development of foldback and induce atrial fibrillation [36].

Previous studies have confirmed that age is the greatest risk factor for atrial fibrillation [37–39]. This is consistent with our results. The reasons for the increased incidence of atrial fibrillation with increasing age are complex. As age increases, remodeling of the atrial myocardium and electrical conduction system occurs, and fibrosis of the interstitium increases [40]. Age-related changes in electrical activity mainly include changes in the shape and duration of cellular action potentials [40], which increase the likelihood of disturbances in the electrical activity of the heart are more likely to occur. An aging myocardium is more susceptible to pathophysiological changes [41]. In addition, aging-related left ventricular diastolic dysfunction, which leads to dilatation and electrical remodeling of the left atrium and pulmonary arteries, also increases the probability of atrial fibrillation [42].

One interesting variable included in our model is PT. Indeed, as of April 2022, we have not found any reports on its association with atrial fibrillation. One possible explanation is that prolongation of PT is caused by the intake of anticoagulant drugs like warfarin, one of the main indications for which is the prevention of thromboembolism due to paroxysmal atrial fibrillation. Therefore, this factor may partly be a sign that the patient has had a previous attack of atrial fibrillation for which they were taking anticoagulants. This is useful when past medical history is not available.

Increased heart rate, especially resting heart rate, is a traditional risk factor for atrial fibrillation [43, 44]. Previous studies have suggested its possible association with disturbance in the autonomic nervous system [45]. The initial sinus heart rate was also included in the model.

Our model also incorporated two critical illness scores, APS III and SOFA, which are used to assess the patient's acute physiological status and organ function, respectively. They are commonly used disease severity scores in the ICU. Atrial fibrillation itself is also an indication of disease severity. Previous studies have demonstrated an association between atrial fibrillation and these scores in various diseases [46, 47].

Our research has several limitations. First, this was a single-center retrospective study using data obtained over a relatively long-time span. In recent years, guidelines for the management of heart failure have been modified, and new drugs and devices have been developed. Second, our study did not include heart rate variability, an indicator of response to sympathetic activity, which may be more closely associated with atrial fibrillation than heart rate [48]. Third, this study failed to include the effect of long-term medication use on arrhythmias in patients. Some patients may have been taking medications such as beta-blockers that reduce the occurrence of atrial fibrillation [49]. Fourth, our nomogram may predict the likelihood of atrial fibrillation in patients with acute heart failure in the ICU, but cannot answer the question of whether prompt intervention in these patients will improve their prognosis. Fifth, the left atrial diameter is an important risk factor for the onset of atrial fibrillation [50, 51]. We did not incorporate echocardiographic measurements into the analysis because the ultrasound reports in the database were unavailable. This might have reduced the accuracy of the model. However, in an ICU setting, echocardiographic results may not be readily available for some patients.

## Conclusion

Our nomogram is effective for predicting the risk of atrial fibrillation in patients with acute heart failure in the ICU. These patients may benefit from early intervention. However, the role of aggressive management in improving prognosis in such patients needs to be assessed in randomized controlled studies.

## Supplementary Information

**Additional file 1:** Development and validation of a nomogram for predicting atrial fibrillation in patients with acute heart failure admitted to the ICU: A retrospective cohort study.

## Data Availability

Due to the license of the MIMIC database, we cannot supply the data file directly. Source code for all analyses can be found at https://github.com/shaou77/af_in_AHF upon publication of this paper, or contact corresponding author Liang Luo (luoliang@mail.sysu.edu.cn).

## Abbreviations

APS III: Acute physiology score III
LASSO: Least absolute shrinkage and selection operator
MIMIC: Medical information mart for intensive care
PT: Prothrombin time
SOFA: Sequential organ failure assessment
ICU: Intensive care unit
CI: Confidence interval
