# Supplementary material for: Development and validation of a nomogram for predicting atrial fibrillation in patients with acute heart failure admitted to the ICU: a retrospective cohort study
Source: BMC Cardiovasc Disord. 2022 Dec 6;22:528. doi: 10.1186/s12872-022-02973-3 (PMC9724334; doi:10.1186/s12872-022-02973-3)
Supplement: Supplementary file 1 — Additional file 1: Development and validation of a nomogram for predicting atrial fibrillation in patients with acute heart failure admitted to the ICU: A retrospective cohort study. [file 12872_2022_2973_MOESM1_ESM.docx]

# Supplementary for :

# Development and validation of a nomogram for predicting atrial fibrillation in patients with acute heart failure admitted to the ICU: A retrospective cohort study

Table S1. The ICD code for ‘acute heart failure’ diagnosis

| ICD code | ICD version | Diagnosis name |
| --- | --- | --- |
| 42821 | 9 | Acute systolic heart failure |
| 42823 | 9 | Acute on chronic systolic heart failure |
| 42831 | 9 | Acute diastolic heart failure |
| 42833 | 9 | Acute on chronic diastolic heart failure |
| 42841 | 9 | Acute combined systolic and diastolic heart failure |
| 42843 | 9 | Acute on chronic combined systolic and diastolic heart failure |
| I5021 | 10 | Acute systolic (congestive) heart failure |
| I5023 | 10 | Acute on chronic systolic (congestive) heart failure |
| I5031 | 10 | Acute diastolic (congestive) heart failure |
| I5033 | 10 | Acute on chronic diastolic (congestive) heart failure |
| I5041 | 10 | Acute combined systolic (congestive) and diastolic (congestive) heart failure |
| I5043 | 10 | Acute on chronic combined systolic (congestive) and diastolic (congestive) heart failure |
| I50811 | 10 | Acute right heart failure |
| I50813 | 10 | Acute on chronic right heart failure |

Table S3 Baseline characteristics of the training group and validation group.

|  | Validation Group | Training Group | *P* |
| --- | --- | --- | --- |
| Number | 701 | 1641 |  |
| Male (%) | 359 (51.2) | 857 ( 52.2) | 0.686 |
| Age (median [IQR]) | 71.59 [60.39, 80.80] | 71.68 [61.57, 81.86] | 0.138 |
| Ethnicity (%) |  |  | 0.285 |
| AMERICAN INDIAN/ALASKA NATIVE | 1 ( 0.1) | 6 ( 0.4) | |
| ASIAN | 16 ( 2.3) | 39 ( 2.4) | |
| BLACK/AFRICAN AMERICAN | 77 (11.0) | 168 ( 10.2) | |
| HISPANIC/LATINO | 15 ( 2.1) | 58 ( 3.5) | |
| OTHER | 36 ( 5.1) | 60 ( 3.7) | |
| UNABLE TO OBTAIN | 8 ( 1.1) | 25 ( 1.5) | |
| UNKNOWN | 101 (14.4) | 209 ( 12.7) | |
| WHITE | 447 (63.8) | 1076 ( 65.6) | |
| Length of hospital stay (day), (median [IQR]) | 11.62 [7.42, 18.17] | 10.97 [7.13, 16.91] | 0.089 |
| Hospital mortality (%) | 101 (14.4) | 191 ( 11.6) | 0.074 |
| Length of ICU stay (day), (median [IQR]) | 4.26 [2.92, 6.97] | 4.12 [2.88, 6.89] | 0.209 |
| Charlson Comorbidity Index (median [IQR]) | 7.00 [6.00, 9.00] | 7.00 [6.00, 9.00] | 0.732 |
| SOFA (median [IQR]) | 6.00 [4.00, 9.00] | 6.00 [3.00, 9.00] | 0.222 |
| APSIII (mean (SD)) | 54.25 (24.48) | 52.82 (23.78) | 0.186 |
| Hematocrit (median [IQR]) | 32.05 [27.80, 37.16] | 32.42 [28.10, 37.45] | 0.148 |
| Mean Blood Pressure (median [IQR]) | 75.04 [69.57, 81.70] | 75.10 [69.16, 82.33] | 0.927 |
| Heart rate (median [IQR]) | 85.06 [74.44, 95.27] | 84.31 [74.06, 95.17] | 0.688 |
| Temperature (℃) (median [IQR]) | 36.79 [36.58, 37.15] | 36.80 [36.56, 37.10] | 0.628 |
| spO2(%) (median [IQR]) | 96.72 [95.15, 98.12] | 96.60 [95.04, 98.11] | 0.584 |
| Respiratory rate (median [IQR]) | 13.00 [10.00, 16.00] | 13.00 [11.00, 16.00] | 0.27 |
| White Blood Cell (*109/L), (median [IQR]) | 11.95 [8.64, 15.51] | 11.70 [8.65, 15.60] | 0.747 |
| Hemoglobin (g/dl) (median [IQR]) | 10.50 [9.05, 12.05] | 10.57 [9.10, 12.25] | 0.329 |
| Albumin(g/dL) (median [IQR]) | 3.30 [2.91, 3.60] | 3.30 [2.90, 3.70] | 0.791 |
| Anion gap (mmol/L) (median [IQR]) | 15.50 [13.50, 18.00] | 15.00 [13.00, 17.50] | 0.233 |
| Hco3 (mmol/L) (median [IQR]) | 23.00 [20.50, 27.00] | 23.00 [20.00, 26.00] | 0.142 |
| BUN (mmol/L) (median [IQR]) | 29.00 [19.00, 48.00] | 27.00 [18.00, 43.00] | 0.016 |
| Creatinine (mmol/L) (median [IQR]) | 1.30 [0.90, 2.10] | 1.25 [0.90, 1.95] | 0.263 |
| Calcium(mmol/L) (median [IQR]) | 8.45 [7.95, 8.90] | 8.40 [8.00, 8.85] | 0.986 |
| Blood Glucose (median [IQR]) | 143.50 [115.50, 185.00] | 142.00 [116.00, 183.50] | 0.878 |
| Sodium (mmol/L) (median [IQR]) | 138.50 [135.50, 140.50] | 138.00 [135.50, 140.50] | 0.189 |
| Potassium (mmol/L) (median [IQR]) | 4.25 [3.90, 4.70] | 4.25 [3.90, 4.70] | 0.935 |
| PT(s) (median [IQR]) | 14.15 [12.60, 16.31] | 13.90 [12.50, 16.40] | 0.331 |
| APTT(s) (mean (SD)) | 45.77 (24.96) | 44.69 (23.90) | 0.337 |
| Alanine aminotransferase(U/L) (median [IQR]) | 36.00 [20.00, 84.00] | 31.00 [17.00, 71.00] | 0.053 |
| Aspartate aminotransferase(U/L) (median [IQR]) | 48.00 [28.00, 128.38] | 45.00 [27.00, 113.50] | 0.331 |
| Troponin-T (ng/ml) (median [IQR]) | 0.29 [0.07, 1.54] | 0.27 [0.07, 1.12] | 0.412 |
| Total bilirubin (median [IQR]) | 0.65 [0.40, 1.10] | 0.65 [0.40, 1.10] | 0.555 |
| NT-proBNP (pg/ml) (median [IQR]) | 5288.00 [1615.00, 14010.00] | 5076.00 [2155.50, 12928.00] | 0.829 |
| Dialysis active in first 24 hours | 24 ( 3.4) | 45 ( 2.7) | 0.447 |
| Urine output (ml/kg) (median [IQR]) | 0.81 [0.47, 1.37] | 0.85 [0.49, 1.38] | 0.359 |
| Vasoactive drug usage in first 24 hours | 316 (45.1) | 714 ( 43.5) | 0.513 |
| Mechanical ventilation in first 24 hours | 320 (45.6) | 722 ( 44.0) | 0.489 |

Table S2 The missing percent of the whole cohort

| Variable | Missing precent % |
| --- | --- |
| Gender | 0 |
| Age | 0 |
| Ethnicity | 0 |
| Length Of Hospital Stay | 0 |
| Hospital Mortality | 0 |
| Length Of ICU Stay | 0 |
| Hematocrit | 0.0539 |
| Hemoglobin | 0.0539 |
| White blood cell | 0.0808 |
| Albumin | 33.01 |
| Anion gap | 0.0808 |
| Hco3 | 0.0539 |
| BUN | 0.0269 |
| Creatinine | 0.0269 |
| Calcium | 2.3444 |
| Blood gas | 0.2425 |
| Na | 0.0539 |
| Potassium | 0.0269 |
| PT | 3.7187 |
| APTT | 4.0151 |
| Alanine aminotransferase | 21.2611 |
| Aspartate aminotransferase | 21.2611 |
| Total bilirubin | 21.3959 |
| Troponin-T | 25.9768 |
| NT-probnp | 46.4026 |
| SOFA score | 0 |
| Heart rate | 0.2156 |
| Mean blood pressure | 0.2156 |
| Respiratory rate | 0.2425 |
| Temperature | 3.4223 |
| Spo2 | 0.2156 |
| Vasoactive drug usage in first 24 hours | 0 |
| APS III | 0 |
| Dialysis active in first 24 hours | 0 |
| Charlson Comorbidity Index | 0 |
| Mechanical ventilation in first 24 hours | 0 |
| Urine output (ml/kg) | 1.6707 |
